# Histone H2A variants play a key role at DNA double-strand breaks during repair pathway choice

**DOI:** 10.3389/freae.2024.1445765

**Published:** 2024-08-22

**Authors:** Emile Clerf, Maxime Galloy, Amélie Fradet-Turcotte, Jacques Côté

**Affiliations:** St-Patrick Research Group in Basic Oncology, Oncology Division of Centre Hospitalier Universitaire (CHU) de Québec-Université Laval Research Center, Laval University Cancer Research Center, Department of Molecular Biology, Medical Biochemistry and Pathology, Faculty of Medicine, Université Laval, Quebec City, QC, Canada

**Keywords:** double-strand break repair, histone H2A variants, chromatin modifiers, post-translational modification, chromatin readers, DNA repair pathway choice

## Abstract

Histone post-translational modifications and variants play crucial roles in the adaptability of chromatin structure, facilitating rapid responses necessary for biological processes such as transcription, replication, and DNA damage signaling. Notably, DNA double-strand break (DSB) signaling heavily relies on these histone modifications, with signal amplification and the recruitment of specific DNA repair factors being dictated by them. Among the histones, H2A and its variants are central to this response, with phosphorylation of the variant H2A.X being the initial and most characteristic histone mark deposit upon DNA damage detection. Additional post-translational modifications of H2A and its variants contribute to the selective recruitment of DNA repair factors and influence the choice of DNA repair pathways. This review provides a summary of current knowledge regarding the roles of histone H2A post-translational modifications and variants in DSB signaling and repair, with a particular emphasis on modifications and variants that impact the choice of repair pathways. Additionally, the involvement of histone chaperones, chromatin modifiers, and remodelers in these processes is discussed.

## Introduction

Preserving genomic integrity is crucial as cells are constantly confronted with multiple DNA-damaging agents from external and internal sources. Among different types of DNA damage, DNA double-strand breaks (DSBs) are the most toxic lesions. Indeed, if they are left unrepaired or not properly repaired, they can lead to mutations, chromosomal aberrations, and even cell death (Khanna and Jackson, 2001). Cells have developed sophisticated mechanisms to signal and repair the damage (Jackson and Bartek, 2009). In response to DSBs, two major repair mechanisms can occur: homologous recombination (HR), which mostly takes place in the late S and G2 phases of the cell cycle, and non-homologous end joining (NHEJ), which can occur throughout the interphase (Ingram et al., 2019). These mechanisms involve distinct actors that favor one mechanism *versus* another to promote a finely tuned signal.

In the nucleus, DNA is found in different levels of compaction depending on the genomic regions. The process of compaction is established by organizing the DNA around the nucleosome, the smallest subunit of the chromatin. It is composed of 147 bp of DNA wrapped around two copies of the four canonical histones (H2A, H2B, H3 and H4) (Luger et al., 1997). In the nucleosome, histones can be modified by post-translational modifications (PTM) via protein complexes called writers, which add chemical groups (methyl, acetyl, phosphate) and/or peptides (Ubiquitin, SUMO) to their N-/C-terminal tails. These marks can be recognized as signals by readers and removed by erasers (Kornberg and Lorch, 2020). Moreover, canonical histones can be replaced by their non-allelic isoforms or histone variants, which give specific properties to the nucleosome and additional versatility in remodeling and signaling events. ATP-dependent remodelers can not only open chromatin by moving or evicting nucleosomes but can also replace specific histones within them (Eustermann et al., 2024). At the heart of this molecular dynamic, histone H2A and its variants play key roles in the response to DNA damage. Furthermore, these histones undergo post-translational modifications regulated and recognized by various molecular effectors through the signaling and repair process.

Specific chromatin modifiers/remodelers increase chromatin dynamics, sliding and/or removing of nucleosomes, allowing accessibility to the DNA, which is required at sites of damages for DNA repair processes (Hauer and Gasser, 2017; Clouaire and Legube, 2019). However, it is still poorly understood in mammalian cells how the specific chromatin remodelers that have been implicated in HR and NHEJ repair mechanisms are recruited and coordinated in their function. Post-translational modifications of H2A and its variants are known to influence chromatin dynamics, implicated in the selection between HR and NHEJ repair mechanisms. These modifications include, phosphorylation by the PI3-kinase-related kinase ATM (*ataxia telangectasia* mutated), ATR (*ATM and RAD3-related*), DNA-PKcs (*DNA-dependent protein kinase, catalytic subunit*), acetylation by histone acetyltransferase such as NuA4/TIP60 (*nucleosome acetyltransferase of H4/Tat-interactive protein, 60 kDa)* and ubiquitylation by enzymes such as RNF8 and RNF168 (*RING nuclear factor*) (Kolas et al., 2007; Doil et al., 2009; Stewart et al., 2009; Mattiroli et al., 2012; Jacquet et al., 2016; Lashgari et al., 2022). In this review, we aim to paint the landscape of the chromatin-modifying events that impact DNA repair pathway choice, and the role of PTMs on H2A and its variants in this process. We will discuss the role of the writer, reader, and chromatin remodeler in these processes. By gathering the current knowledge, we will present the underlying mechanisms that orchestrate DNA signaling and repair. This systematic review of current literature may unveil avenues for exploring and developing precise strategies to address genetic diseases and cancer that are driven by unproper repair of DNA breaks.

### The control of DSB repair choice

The DSB repair pathway choice is first dictated by the phase of the cell cycle (Hustedt and Durocher, 2017). HR is restricted to the S and G2 phases, implicating DNA end resection and homology search, as this process uses the newly synthesized double helix from the sister chromatid as a template to repair the damaged one, generally producing accurate repair product (Aylon et al., 2004; Ira et al., 2004). NHEJ, on the other hand, is a DSB repair mechanism that requires few or no processing as it brings together DNA ends by a multi-protein synaptic complex to directly ligate them (Lieber, 2010). This pathway can occur throughout interphase. HR and NHEJ are mutually exclusive, and disruption of either of the pathways sensitizes cells to DSB-inducing drugs and results in spontaneous chromosomal aberrations (Takata et al., 1998; van de Kooij et al., 2022). Thus, the first steps of the cellular response to DNA damage include the selection of the specific repair pathway.

Controlling the initiation of one repair pathway at the expense of the other ensures intrinsic genome integrity. Initiation of DSB repair by HR during the G1 phase can lead to aberrant repair using a template that is weakly homologous to the damaged sequence or fully random (Kasparek and Humphrey, 2011). Furthermore, NHEJ, if initiated in the G2 phase, disadvantages HR and the opportunity to repair DNA with high fidelity (Fugger and West, 2016; Choi et al., 2020). Thus, the DSB repair pathway choice is key in maintaining genome integrity and this choice is a dynamic process involving the recruitment of opposing factors at the DSB (anti-resection factors vs resection initiator) and the surrounding chromatin (Hustedt and Durocher, 2017; Sigismondo et al., 2023). Failure to properly repair DSBs can lead to pathological genomic alterations (Ceccaldi et al., 2016).

### The early response to DSBs is shared by NHEJ and HR pathways

The initial cellular response to DSBs shares common signaling events between repair pathways. Following a double-strand break, the MRN protein complex, consisting of MRE11 (*Meiotic Recombination 11*), RAD50 and NBS1 (*Nijmegen breakage syndrome 1 mutated*), is recruited to the lesion, forming a synapse point, i.e., holding DNA ends in close proximity (Rass et al., 2009; Reginato and Cejka, 2020; Rotheneder et al., 2023). This event leads to the activation of the PI3-kinase-related kinase (PIKK) ATM that phosphorylates the histone H2A variant H2A.X at its C-terminus (S139) in chromatin surrounding the break (∼1 Mb), as well as proteins such as MDC1 (*Mediator of DNA damage checkpoint protein 1*) and MRE11 (Burma et al., 2001; Collins et al., 2020). Next, the E3 ubiquitin ligase RNF8 (RING finger protein 8) is recruited to the breakthrough the recognition of ATM-dependent phosphorylation of MDC1. RNF8 poly-ubiquitylates either histone linker H1 or L3MBTL2 (*lethal(3)malignant brain tumour-like protein 2*), the latter being also recruited by MDC1 at sites of damage (Huen et al., 2007; Thorslund et al., 2015; Nowsheen et al., 2018). This K63 poly-ubiquitin chains enable the subsequent recruitment of the E3 ubiquitin ligase RNF168 (*RING finger protein 168*) that ubiquitylates lysines 13 and 15 of histone H2A (H2AK13 or K15ub) and its variants (Mattiroli and Penengo, 2021; Kelliher et al., 2022). In parallel, depending on the cell cycle stage and genomic location, lysine 20 of histone H4 is unmodified, mono-, di- or tri-methylated (H4K20me0, me1, me2 or me3) (Mattiroli and Penengo, 2021). In the section below, we will discuss further the importance of this methylation state in the implementation of the repair pathway choice, either NHEJ or HR (Becker et al., 2021) as well as its crosstalk with H2A modifications at DSBs. Ultimately, the critical determinant of the repair pathway choice is whether a DSB undergoes 5' → 3′ resection, which is required for HR, while the resulting 3′ ssDNA overhang is a poor substrate for the NHEJ (Reviewed in: (Ceccaldi et al., 2016; Scully et al., 2019)) (Figure 1).

**FIGURE 1:**
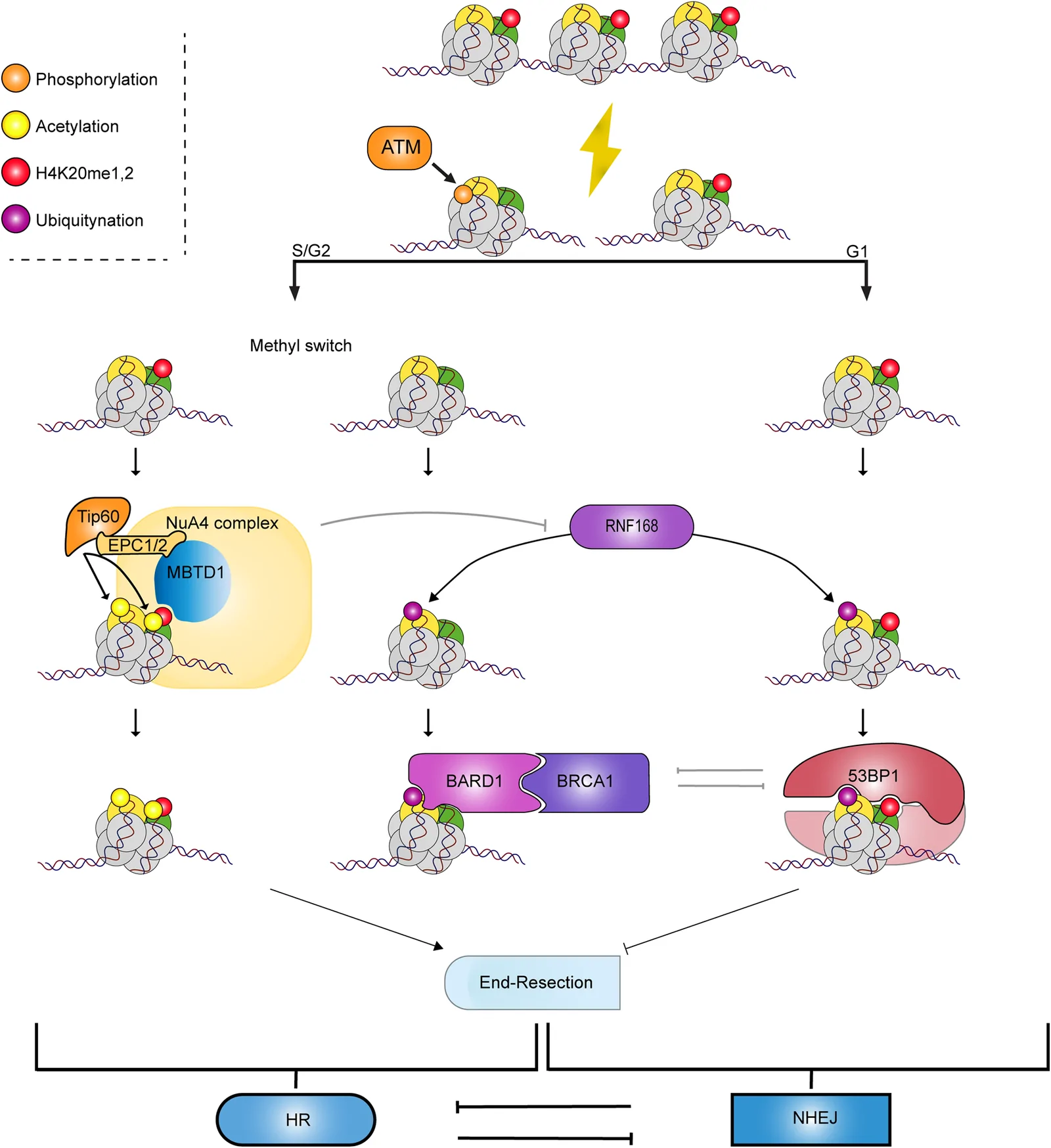
Crosstalk between H2A and H4 modifications to recruit chromatin readers to DNA breaks. When DSB occurs, MDC1 binds to H2A.X, which is phosphorylated by ATM (γH2A.X). ATM’s phosphorylation of MDC1 leads to the recruitment of the E3 ubiquitin ligase RNF8 and RNF168. Depending on the methylation status of H4K20, different repair effectors are recruited, leading to the two main DSB repair pathways, which are mutually exclusive: HR mediated by the BARD1/BRCA1 complex and NHEJ through 53BP1.

### Commitment to the NHEJ pathway

In the context of these chromatin modifications, NHEJ initiation is characterized by the recruitment of 53BP1 (*p53 Binding Protein 1*) through the collaboration of its oligomerization domain, its UDR (*Ubiquitin-dependent recruitment*) and its tandem Tudor domains in recognizing nucleosomal H2AK15Ub and H4K20me2 (Fradet-Turcotte et al., 2013). 53BP1 is then bound by RIF-1 (*Replication Timing Regulatory Factor 1)*, and this interaction allows the recruitment of the Shieldin complex, composed of SHLD1, SHLD2, SHLD3 (*Shieldin Complex Subunit 1-2-3*) and REV7 (also known as *MAD2L2*) (Setiaputra and Durocher, 2019). The Shieldin complex is composed of two functional modules. The first one is SHLD1/2, which is a localization module, and the second one is SHLD3/REV7, which is the effector domain of the complex interacting with RIF1 (Gupta et al., 2018; Ghezraoui et al., 2018). The Shieldin complex appears to be a central component of NHEJ, protecting the DNA from the 5' → 3′ exonucleases activity, a critical step in the processing of the DNA ends during the early phases of HR (Mirman et al., 2018).

NHEJ is led by the proteins Ku70/80 that bind the broken ends, 20 to 200 bp of DNA flanking the damaged site (Walker et al., 2001; Zhang et al., 2001) and block resection. Ku70/80 interact with the DNA sugar backbone, ignoring specific DNA sequences (Downs and Jackson, 2004). The crystallographic structures of Ku70/80 present the heterodimer as a scaffolding complex allowing the recruitment of the PIKK-family DNA-PKcs. Ku70/80 heterodimer and DNA-PKcs forms DNA-PK, which serves as a platform that controls DNA-end sensing, protection and processing, before pairing and ligation (Jette and Lees-Miller, 2015). Then, the autophosphorylation of DNA-PKcs loosens the DNA-PK interaction with the DNA and restrains Artemis nuclease activity to avoid excessive DNA-end processing. This allows the recruitment of NHEJ factors XRCC4 (*X-ray repair cross-complementing protein 4)*, XLF (*RCC4-like factor)* factors, ligase IV and the APLF (*Aprataxin and PNKP like factor*) nuclease to process and ligate DNA ends (Rivera-Calzada et al., 2007; Liu et al., 2022). This complex enables the two DNA ends to be clamped to prevent rearrangements and chromosomal aberrations and the two non-homologous ends to be ligated by ligase IV (Chang et al., 2017).

### Commitment to the HR pathway

In the S/G2 phase, a DSB can be repaired using homologous recombination. This process uses the available sister chromatid as a template for repair. Resection of DNA-ends at the DSB generates a 3′ ssDNA overhang that invades the homologous chromatid to faithfully repair the damage (Jasin and Rothstein, 2013). The chromatin state surrounding the DNA breaks acts as a signaling hub by recruiting or inhibiting the recruitment of repair factors to direct repair to HR. HR is initiated by default in the absence of 53BP1, a DNA repair factor that guides repair toward NHEJ by preventing resection (Bunting et al., 2010). Indeed, knocking out 53BP1 in BRCA1 (*Breast Cancer gene 1*)-deficient cells rescues resection and subsequent homologous recombination (Bouwman et al., 2010). As previously mentioned, 53BP1 is recruited to chromatin ubiquitylated on H2AK15 and di-methylated on H4K20. However, during the S phase, newly synthesized histone H4 are unmethylated, which dilutes the presence of H4K20me1/2 from the parental nucleosome and concomitant recruitment of 53BP1 (Saredi et al., 2016; Escobar et al., 2021; Stewart-Morgan et al., 2023). H4K20 methylation status is thus important during the DSB repair pathway choice. The absence of 53BP1 on the nucleosome due to the absence of H4K20me1/2 allows the interaction of the BARD1 (*BRCA1-associated RING domain protein 1*)-BRCA1 complex with the nucleosome via the BARD1 ARD and BRCT domains that bind to H4K20me0 and H2AK15Ub, respectively (Nakamura et al., 2019; Becker et al., 2021; Dai et al., 2021; Hu et al., 2021; Witus et al., 2022; Burdett et al., 2023). After replication, H4K20 is rapidly mono-, di- or trimethylated by SET8 and SUV420H1/2, respectively (Pesavento et al., 2008; Jørgensen et al., 2013). In parallel, the H4K20me1/2 can be recognized by the MBTD1 (*mbt (malignant brain tumor) domain containing 1*) subunit of the NuA4/TIP60 acetyltransferase complex. Tip60 then acetylates lysines on the H2A and H4 tails, which hinders the interaction of 53BP1 with H4K20me1/2 (Tang et al., 2013; Jacquet et al., 2016; Lashgari et al., 2022). BARD1 promotes resection of the terminal ends via ubiquitylation of the endonuclease CtIP (*CtBP-interacting protein*) (Barber and Boulton, 2006). At the break, the heterodimer BRCA1/BARD1 poly-ubiquitylates residues K125, K127 and K129 of nucleosomal H2A (Kalb et al., 2014). Evidence showed that inhibiting this ubiquitylation, through USP48 (*Ubiquitin specific peptidase 48*) activity, restrains resection and consequently homologous recombination (Uckelmann et al., 2018). The ubiquitylation of H2A C-terminal residues allows end resection by EXO1 (*Exonuclease 1*) and DNA2/BLM (*Bloom syndrome protein*) helicase activities. The free 3′ ends are then rapidly coated by RPA (*Human Replication protein A*), a key protein in protecting the single-stranded DNA from degradation. RAD51 is subsequently loaded onto the DNA, replacing RPA. RAD51 loading involves BRCA2 in complex with PALB2 (*Partner and localizer of BRCA2*) (Davies et al., 2001; Ducy et al., 2019). RAD51 bound to the long overhang ssDNA forms a synapse to browse chromosomes. Once the search for homology is done, RAD51 opens the double helix and invades the complementary strand forming the D-loop through ATP hydrolysis, positioning of the damaged DNA at the homologous site (van der Heijden et al., 2008; Wald et al., 2022). This event recruits polymerases such as Pol δ that will fill the gap caused by the damage (Li et al., 2009). The displacement of the D-loop occurs through different sub-pathways of HR such as Single-Strand Annealing (SSA), Holliday junction HR (dHR) or Break-Induced Replication (BIR) (reviewed by Piazza and Heyer (Piazza and Heyer, 2019)) leading to the recovery of the genetic integrity at the site.

The chromatin landscape surrounding the break sites is directly involved in the whole process of repair. Nonetheless, the dynamic role of the different histone PTMs is still only partly understood and need to be further studied in the context of the repair pathway selection. Therefore, we gathered relevant knowledge related to H2A, its variants and their post-translational modifications after DSB that lead to the repair pathway choice.

### The histone H2A family

The histone H2A family is the most heterogeneous family of histones (Henikoff and Smith, 2015). H2A has numerous variants expressed in most cell types, such as H2A X, H2A Z.1, H2A Z.2 and macroH2A. H2A variants are encoded by paralogous genes harboring sequences that are highly conserved with canonical H2A but differ in their N- and C-termini.

The presence of H2A variants increases the diversity of the chromatin landscape and modulates the signals to various chromatin-modifying players such as RNF168, TIP60/NuA4 and BRCA1/BARD1. The inherent properties of those variants and their modifications refine the recruitment of key factors and the structure of chromatin itself during DSB signaling and DNA repair pathway choice. The choreography of the writing, erasing, remodeling, and reading proteins along DSB signaling is determinant during the choice of the repair pathway. Histone H2A and its variants are thus key elements in the regulation of repair pathway choice (Figure 2).

**FIGURE 2:**
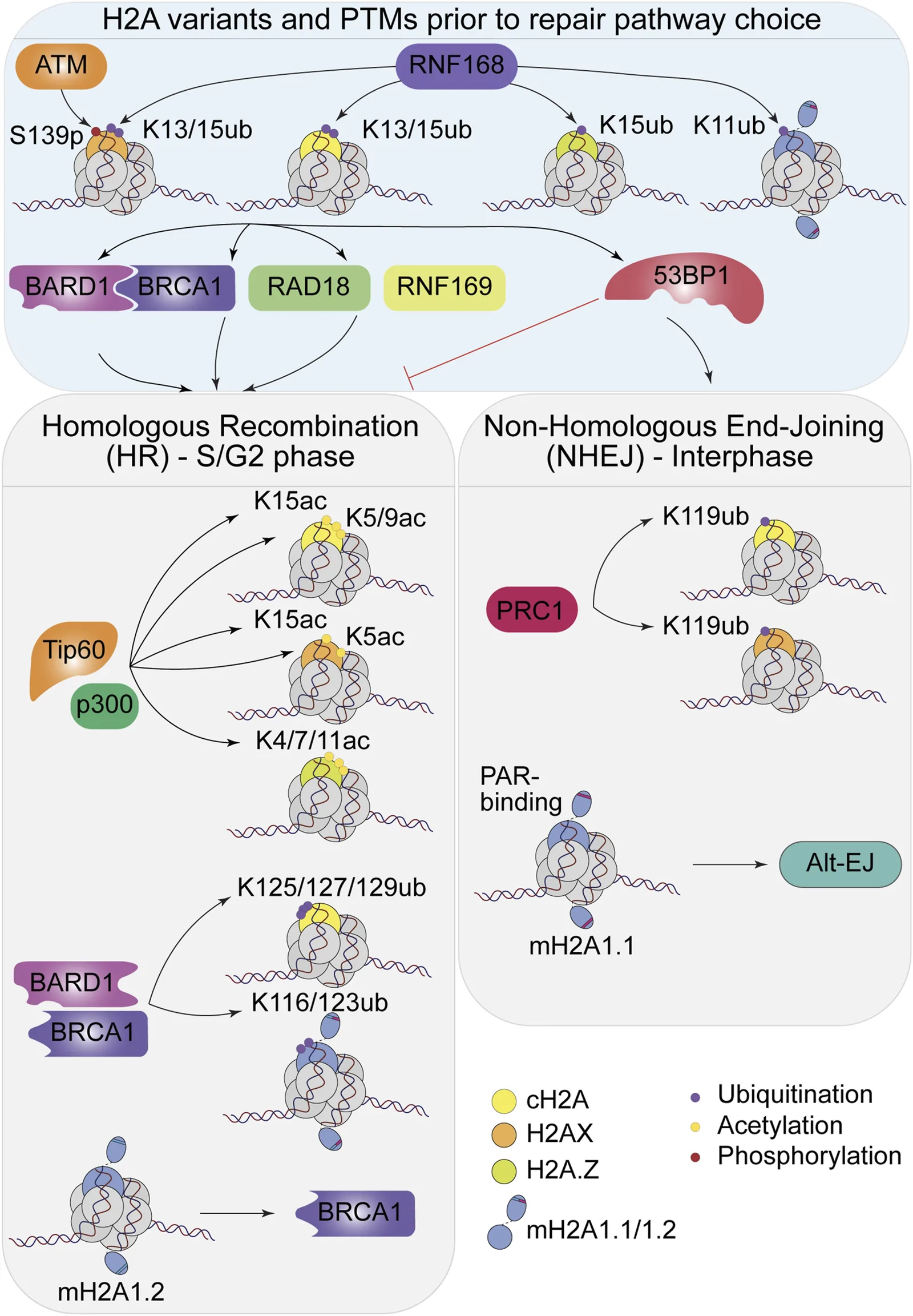
H2A variants and their PTMs impact the repair pathway choice. Top: Modifications happening prior to the choice, by ATM and RNF168 to recruit the DSB repair factors. Bottom: Choice between HR and NHEJ mediated by the repair factors, impact of the H2A variants and their PTMs.

### The canonical H2A

We previously discussed the role of the ubiquitylation of H2AK13/15 by RNF168 (Mattiroli et al., 2012) during DSB signaling, but several other modifications on this canonical histone also impact the outcome of repair. H2A lysine 119 is mono-ubiquitylated by the E3 ubiquitin ligase BMI1/RING1B, part of the Polycomb repressive complex 1 (PRC1), to prevent *de novo* transcription at damage sites (Ismail et al., 2010; Ginjala et al., 2011; Tamburri et al., 2020). Indeed, this mark is found on repressed genes, within H3K27me3-containing regions, deposited by the PRC2 methyltransferase complex (Schwartz and Pirrotta, 2013; Tamburri et al., 2020). Both PRC1 and PRC2 can be recruited to DSBs leading to neighboring gene repression to prevent conflict between transcription and the DNA repair machineries. Thus, H2AK119ub plays a role in transcription silencing in the damaged chromatin (Campbell et al., 2013). Additionally, BMI1-mediated mono-ubiquitylation of H2AK119 facilitates the recruitment of CtIP, promoting DNA end resection and allowing recruitment of key factors of the HR pathway, such as RPA, BRCA1 and RAD51 at DNA damage sites (Fitieh et al., 2022). In parallel, BAP1 (*BRCA1-associated protein 1*), the deubiquitinase of H2AK119, is phosphorylated in an ATM-dependent manner in response to DNA damage and is also required for efficient HR repair, highlighting the importance of highly dynamic H2AK119ub turnover at sites of DNA damage (Ismail et al., 2014). Canonical H2A possesses other lysines in its C-terminus, K125, K127 and K129. Interestingly, NMR studies revealed that these residues can be ubiquitylated by the heterodimer BRCA1/BARD1 (Witus et al., 2022). The E3 ligase is required for several functions of this heterodimer during DSB signaling (Barber and Boulton, 2006). Among them, ubiquitylation of H2AK125/127/129 recruits the ATP-dependent chromatin remodeler SMARCAD1 (*SWI/SNF-related, matrix-associated actin-dependent regulator of chromatin*), via its ubiquitin-binding CUE (*coupling of ubiquitin conjugation to ER degradation*) domains. SMARCAD1 is essential for nucleosome sliding and eviction at damaged sites, but also in 53BP1 repositioning, helping DNA end resection and processing with the CtIP nuclease (Densham et al., 2016; Sadek et al., 2022). Interestingly, loss of the specific deubiquitinase (DUB) for BRCA1/BARD1-dependent H2AK125/127/129ub, USP48, increases DNA end resection and RAD51 foci formation. USP48 thus serves as an antagonist of BRCA1 and limits RAD51 accumulation to ensure genomic stability (Uckelmann et al., 2018). Note that the BRCA1-BARD1 complex also ubiquitylates non-histone substrates such as CtIP (Yu et al., 2006; Wang et al., 2021; Bolck et al., 2022), highlighting the multifaceted role of proteins that act as histones writers and processing enzymes during initiation of the repair pathway.

Importantly, it has been very recently shown that canonical H2A di-acetylation on lysine 5 and 9 (K5/K9) by NuA4/TIP60 and p300 during the early stages of DSB signaling can create a non-permissive state of chromatin, impeding the NHEJ-end synapsis, thus promoting HR. Indeed, deacetylation of H2AK5/9ac by the mitotic deacetylase complex (MiDAC) suppresses hyper-accumulation of bromodomain-containing protein BRD4, which prevents proper DNA end synapsis, key to efficient NHEJ repair (Bao et al., 2024).

### H2A.X, the variant signaling repair

Upon detection of DSBs, activation of PIKKs ATM, ATR and DNA-PKcs lead to the addition of the first DSB-induced histone mark by phosphorylating the H2A.X variant at serine 139 (S139) (Rogakou et al., 1998), known as γH2AX. The phosphorylation of S139 of H2A.X is dispensable for the recruitment of DNA damage repair elements (Celeste et al., 2003) but its absence decreases the recruitment of BRCA1 and RAD51, two effectors of the DNA repair by HR (Bassing et al., 2002), and increases chromosomal aberrations leading to higher risk of tumor development in *H2AX* null mice (Celeste et al., 2002). Phosphorylation of H2A.X triggers a series of downstream modifications, being bound by MDC1 that is also phosphorylated by ATM and leading to the recruitment of RNF8. RNF8 catalyzes poly-ubiquitylation (K63 chain) of histone linker H1 or the protein L3MBTL2 (Huen et al., 2007; Kolas et al., 2007; Thorslund et al., 2015; Nowsheen et al., 2018), which is recognized by RNF168. The latter E3-ubiquitin ligase then mono-ubiquitylates lysines 13 and 15 (K13, K15) on histone H2A and H2A.X (Gatti et al., 2012; Mattiroli et al., 2012). Besides the phosphorylation of H2A.X on S139, the acetylation of H2A.X plays multiple roles in damage signaling. The acetylation of lysine 5 by Tip60 promotes the accumulation of NBS1 at double-strand breaks (Ikura et al., 2015). Indeed, loss of H2A.X acetylation at K5 leads to dispersed signal and improper accumulation of NBS1 onto damaged chromatin and increases γH2AX foci after DNA damage (Ikura et al., 2015). Conversely, Tip60 also modifies K15 on H2A and H2A.X, the same target as RNF168 required for the recruitment of 53BP1 and promotion of NHEJ repair (Jacquet et al., 2016). In the model proposed by *Jacquet et al.,* acetylation of the K15 by the NuA4/TIP60 complex blocks its ubiquitylation by RNF168, promoting HR over NHEJ (Fradet-Turcotte et al., 2013; Jacquet et al., 2016). However, a more recent model revealed that H2AK15ub also contributes to HR repair by driving element for repair pathway choice that comes from the methyl switch of H4K20 (Becker et al., 2021). The interplay for the accessibility and modification of H2AK15 in combination with H4K20 modification status still raises many questions and additional studies will be needed to define when and where H2AK15 is acetylated during cell cycle.

In addition to DNA repair protein, phosphorylation of H2A.X contributes to the recruitment of remodeling complexes SWI/SNF and INO80 through direct recognition of the modified histone at DSBs (Morrison et al., 2004; van Attikum et al., 2004; Park et al., 2006; Ogiwara et al., 2011; Harrod et al., 2020). While INO80 promotes resection, the current literature supports a role of SWI/SNF during strand invasion. Further studies will be required to characterize the role of these remodelers in DNA repair.

Interestingly, the NuA4/TIP60 complex has also been linked to the remodeling of H2A.X chromatin through an acetylation-linked degradative poly-ubiquitylation as well as its SWI/SNF-family ATPase subunit p400 increasing chromatin accessibility for repair mechanisms (Ikura et al., 2007; 2015; Xu et al., 2010; Courilleau et al., 2012). In the yeast model, γH2A is removed from chromatin surrounding the break to allow resection and this implicates the initial action of NuA4 followed by remodelers (Cheng et al., 2018; Cheng et al., 2021). γH2A can also be replaced by the H2A variant H2A.Z, modulating the DNA repair signaling (Downs et al., 2004; van Attikum et al., 2007; Kalocsay et al., 2009; Horigome et al., 2014; Lee et al., 2014; Van et al., 2015). Moreover, NuA4/TIP60 subunit p400 catalyzes the exchange of γH2Av for H2Av in *D. melanogaster* (Kusch et al., 2004). H2Av is the fly ortholog of both H2A.X and H2A.Z, containing the SQ*Y domain at its C-terminus, which can be phosphorylated by ATM near DSBs, while its N-terminus and acidic patch is similar to H2A.Z (Bonisch and Hake, 2012). It has been suggested that the mammalian NuA4/TIP60 complex could remove γH2AX from the chromatin at DSBs and load H2A.Z (Altaf et al., 2010; Xu C. et al, 2012), as discussed further in the next section. For γH2AX to be able to recruit MDC1, its tyrosine 142 needs to be dephosphorylated by EYA (*Eyes absent*). This process is timely regulated by ZNF506 (*Zinc finger protein 506*) and allows the recruitment of downstream repair factors that amplify the repair signal (Cook et al., 2009; Nowsheen et al., 2018).

Among residues targeted for ubiquitylation in canonical H2A, K13, K15 and K119 are conserved in H2A.X and ubiquitylated upon the detection of DSBs (Pan et al., 2011; Wu et al., 2011; Mattiroli et al., 2012). H2A.X N-terminus is acetylated at its lysine K36 by CBP/p300 acetyltransferases, and this modification is required for cell survival after exposure to DNA damage, independently of γH2AX (Jiang et al., 2010). Indeed, mutants of K36 and T101, a site that is phosphorylated, lead to IR sensitivity, but rescued HR efficacy in *H2AX*−/− mouse ES cells at the same level that wild type H2A.X (Xie et al., 2010). This means that these residues contribute to IR resistance by a new, yet undiscovered, repair mechanism.

Elimination of γH2AX from the chromatin is essential for cell cycle progression after DSB repair (Fernandez-Capetillo et al., 2002; Nakamura et al., 2004; Shibata et al., 2010). Phosphatases PP2C and PP4 appear to be relevant candidates for this task (Chowdhury et al., 2005; Keogh et al., 2006; Nakada et al., 2008). Nonetheless, the question of whether dephosphorylation occurs on chromatin or after removal is still debated.

### H2A.Z, a key regulator of chromatin dynamics

Histone variant H2A.Z is ∼60% homologous with canonical H2A. H2A.Z has more positive charged amino acids in its N-terminal tail, including two additional lysines, leading to a proposed intrinsic stabilization/destabilization of the nucleosome depending on the acetylation status (Abbott et al., 2001; Zhang et al., 2005; Zlatanova and Thakar, 2008; Draizen et al., 2016) (Figure 2). This variant also contains a more extended acidic patch, which is involved in regulating higher-order chromatin formation (Fan et al., 2004). H2A.Z is essential for the development and survival of mice as its depletion in mice leads to growth and metabolism defects (Belotti et al., 2024). H2A.Z is also a target of RNF168, demonstrated by *in vitro* and *in cellula* assays (O’Connor et al., 2015). The ubiquitylation profile of H2A.Z shows di-ubiquitylation like H2A and H2A.X. Lysine 15 of H2A.Z is the required first ubiquitylated residue, allowing ubiquitylation of other lysines (Fradet-Turcotte et al., 2013; Kelliher et al., 2020). Unfortunately, the identity of the other modified lysine(s) is not known. H2A.Z C-terminus is ubiquitylated and this modification has been associated with gene repression similar to canonical H2A (Sevilla and Binda, 2014). Reminiscent of canonical H2A, the C-terminal tail of H2A.Z is a substrate of the E3 ubiquitin ligase RING1B, and ubiquitylation of the K120, K121 and K125 residues is found at transcriptionally silent heterochromatin on the inactive X chromosome (Sarcinella et al., 2007).

Acetylation of H2A.Z is a key modification that occurs at the transcription start site (TSS) of active genes and is associated with epigenetic gene deregulation in tumorigenesis (as reviewed in (Giaimo et al., 2019). H2A.Z acetylation colocalizes with γH2AX at DSB (Hayakawa et al., 2017). K4, K7, K11 and K13 can be acetylated, playing a role in transcription, but di- or tri-acetylation is also observed in the context of DNA repair, conferring nucleosome destabilization and an open conformation (Colino-Sanguino et al., 2022). Loss of K7 acetylation, in TIP60-depleted cells, is sufficient to induce a p53-independent cell death (Wichmann et al., 2022). ChIP-seq mapping of chromatin composition/modifications after double-strand breaks showed different compositions of histone variants and PTMs depending on the genome location and the chosen repair pathway. It appears that total H2A.Z is significantly diminished during repair by HR, whereas H2A.Zac is unchanged, suggesting that ratio of H2A.Z acetylation greatly increases during HR (Taty-Taty et al., 2014; Clouaire et al., 2018). The genomic mapping approach demonstrated no significant changes of H2A.Z or H2A.Zac on chromatin during NHEJ repair (Clouaire et al., 2018). Besides, in the yeast model, SUMOylation of H2A.Z can lead to the recruitment of RAD51 (Kalocsay et al., 2009). Altogether, these data support a role of H2A.Z in the HR repair pathway. Nonetheless, knock-down/out H2A.Z cell lines show defective recruitment of Ku80 and downstream elements of NHEJ repair pathway (Belotti et al., 2024) as well as BRCA1 at damage sites (Xu C. et al, 2012).

H2A.Z has in fact two isoforms, H2A.Z.1 and H2A.Z.2, encoded by two different genes, that differ by only 3 amino acids (Kreienbaum et al., 2022). Studying specifically each isoform/amino acid variation may be key for a better understanding of H2A.Z involvement in the different repair pathways, as suggested by a recent study (Chen et al., 2023). It is known that the NuA4/TIP60 complex is a player in the deposition of H2A.Z and data on endogenous H2A.Z shows that NuA4/TIP60 has a preference for H2A.Z.2, while SRCAP, the other remodeler responsible for H2A.Z deposition, does not show such bias (Lamaa et al., 2020). Interestingly, it has been suggested that H2A.Z can serve as a recruitment platform for SUV420H1 to di-methylate H4K20, a crucial mark for 53BP1 binding and orientation towards the NHEJ repair pathway (Abini-Agbomson et al., 2023; Huang et al., 2023). Unfortunately, both H2A.Z isoforms are targeted in most studies, or only H2A.Z.1. The mechanism of H2A.Z removal from chromatin is controversial, but it has been shown that chaperone ANP32E (*Acidic nuclear phosphoprotein 32 family member E*) can remove H2A.Z from intact nucleosomes and is rapidly recruited to DSBs (Gursoy-Yuzugullu et al., 2015). It was suggested to promote H4 acetylation, reshape the local chromatin and facilitate DNA repair. Loss of ANP32E leads to increased end-resection by CtIP, creating single-stranded DNA and increased repair by alternative NHEJ (Alt-EJ) (Gursoy-Yuzugullu et al., 2015). In parallel, the INO80 chromatin remodeler has been suggested to regulate H2A.Z dynamic in chromatin (Papamichos-Chronakis et al., 2011). INO80 is the first remodeler that has been shown to play a role at DSBs, assisting end resection by remodeling chromatin (Downs et al., 2004; Morrison et al., 2004; Van Attikum et al., 2004). Obviously, significant work is still needed to dissect H2A.Z dynamics and function at DSBs and how it can influence repair pathway choice.

### macroH2A, the largest histone

MacroH2A differs from other H2A variants by its distinct non-histone domain composed of a disordered linker region and a globular macrodomain. Three isoforms of macroH2A are produced from genes *H2AFY* and *H2AFY2*, also known as *MACROH2A1* and *MACROH2A2,* respectively (Pehrson and Fried, 1992; Costanzi and Pehrson, 2001). While only one mRNA is produced from *MACROH2A2, MACROH2A1* mRNA is alternatively spliced to produce macroH2A1.1 and macroH2A1.2 isoforms (Pehrson et al., 1997). Splicing occurs between two mutually exclusive variants of exon 6 of *MACROH2A1* mRNA, making isoforms that differ by only 33 amino acids within the macrodomain. This difference renders macroH2A1.1 capable of binding to poly (ADP-ribose) (PAR) (Karras et al., 2005; Kozlowski et al., 2018), an interaction that is essential for its role in the repression of transcription and DNA damage signaling on chromatin (Timinszky et al., 2009; Sebastian et al., 2020). MacroH2A1.2 and macroH2A2 show no affinity for PAR moieties, and no interactors of the macrodomain have been identified for macroH2A1.2 (Kustatscher et al., 2005; Kozlowski et al., 2018).

Structural studies show that macroH2A preferentially assembles into hybrid macroH2A-H2A nucleosomes in which protein-DNA and protein-protein interactions are more stable (Chakravarthy and Luger, 2006; Bowerman et al., 2019). MacroH2A is specifically incorporated in the chromatin by the LSH/HELLS (*Lymphoid-specific helicase/Helicase lymphocyte specific*) remodeler in an ATP-dependent manner, both *in vitro* and in cells (Ni et al., 2020; Ni and Muegge, 2021). In human genome, macroH2A accumulation is enriched at loci that exhibit high levels of replication stress, such as the common fragile sites (Kim et al., 2018) and at telomeres in cells where their replication is dependent of alternative lengthening of telomere (ALT) (Kim et al., 2019). Consistently, LSH/HELLS-depleted cells are prone to replication fork stalling and exhibit increased levels of DNA damage, a phenotype that is similar to the one observed in macroH2A-depleted cells (Kim et al., 2018; Xu et al., 2021). Depletion of LSH/HELLS, macroH2A1 or macroH2A2 lead to a reduction of RAD51 filament formation at stalled replication forks and to concomitant nuclease-dependent degradation of unprotected forks (Xu et al., 2021). Improper accumulation of 53BP1 at stalled forks contributes to this reduction as this phenotype is rescued by 53BP1 depletion in LSH/HELLS-deficient cells. Note that Xu et al., 2021 also report LSH/HELLS- and macroH2A1.2-dependent change in histone PTM at stalled forks (H3K4me3 and H4K20me2), suggesting that macroH2A loading at stalled replication forks impacts the chromatin environment. Other chaperones contribute to the efficient loading of macroH2A on chromatin as depletion of the histone chaperone complex associated with the replicative helicase, FACT (*FAcilitates Chromatin Transcription*), also impairs the deposition of macroH2A1.2 at sites of replication stress (Kim et al., 2018). In ALT cells, macroH2A1.2 incorporation at sites of replication stress is dependent on the chromatin remodeler ATRX, which negatively regulates the amount of macroH2A at telomeres (Ratnakumar et al., 2012; Kim et al., 2019).

MacroH2A1.2 and macroH2A1.1 were both originally described to accumulate on the mammalian inactive X chromosome (Xi) and promote the formation of the Xi macro-body (Costanzi and Pehrson, 1998). However, the role of macroH2A1.2 as a “replication-stress”-protective histone variant was recently shown to be important for the integrity of the Xi and associated female mice survival (Sebastian et al., 2020). At this locus, macroH2A1.2 actively counteracts Xi-specific anaphase defects and chromosomal instability that are induced by a macroH2A1.1-driven activation of Alt-EJ and thus, improper DNA repair pathway choice.

MacroH2A1.2 accumulates at the I-SceI-induced DSBs in an ATM-dependent manner (Khurana et al., 2014), while macroH2A1.1 accumulates in a PARP1-dependent manner at sites of damage (Timinszky et al., 2009; Xu Y. et al, 2012). The roles of macroH2A isoforms and their remodelers in DSBs signaling and repair have been studied using DNA repair reporter assays. In these assays, depletion of macroH2A1.2 reduces HR efficiency in a 53BP1-dependent manner, coherent with a model where macroH2A1.2 favors the recruitment of BRCA1 at DSBs (Khurana et al., 2014). A similar phenotype is observed at telomeres in ALT cells (Kim et al., 2019), a process that heavily relies on homology-driven repair. In the latter conditions, the depletion of macroH2A1.2 reduces the recruitment of BRCA1 at the telomeres and safeguards their genomic stability. In Khurana et al., 2014, depletion of the H3K9 methyltransferase PRDM2 also rescues the levels of HR, with no further impact upon co-depletion of macroH2A1. These findings suggest that, in addition to its role in regulating the recruitment of BRCA1, macroH2A’s impact on DNA repair pathway choice at DSBs also relies in part on the compaction of the chromatin surrounding the break. While macroH2A1.2 promotes HR, efficient NHEJ repair has been associated with the PAR-binding capacity of macroH2A1.1 (Timinszky et al., 2009; Xu Y. et al, 2012; Sebastian et al., 2020). The mechanism by which macroH2A participated in the recruitment of NHEJ factors is unclear. At DNA break induced by laser stripes, the recruitment of GFP-Ku80 is reduced in LSH/HELLS knocked-out cell lines. Consistent with a role of macroH2A in NHEJ, depletion of LSH/HELLS reduces NHEJ levels in DNA repair reporter assays (Unoki et al., 2018). Other groups reported that accumulation of GFP-Ku70 at laser stripes is reduced in cells expressing macroH2A1.1, suggesting an inhibitory role in this condition (Timinszky et al., 2009). A possible explanation could be an indirect impact of macroH2A1.1 ectopic expression on chromatin compaction. Indeed, both the compaction of the chromatin and the interaction of macroH2A1.1 with DNA repair factors such as PARP1, LIG3 and XRCC1 have been reported, making the dissociation of the two functions complex. Note the ectopic expression of macroH2A1.1 also leads to improper pathway choice by promoting Alt-EJ, a phenotype that may contribute to the acquisition of genomic instability in tumor cells (revised in (Oberdoerffer and Miller, 2023)).

As canonical histone H2A, macroH2A isoforms are targeted by histone-modifying enzymes in response to DNA damage. Co-transfection of Myc-RNF168 with SFB-macroH2A1 in HEK293T cells leads to the ubiquitylation of macroH2A1 (Kelliher et al., 2020). The ubiquitylation of macroH2A by RNF168 may offer additional docking platforms for recruiting DNA repair proteins to DNA damage. Similarly to what has been shown for H2AK129, the heterodimer BRCA1/BARD1 also ubiquitylates macroH2A on K123 (Kalb et al., 2014; Kim et al., 2017). Mutations of this site in cells lead to defective replicative senescence (Kim et al., 2017). However, a role for this modification in DNA repair is not excluded, as hypothesized for the ubiquitylation of canonical H2AK125/127/129 by BRCA1, further investigations are, nonetheless, required to clear this hypothesis. Interestingly, mass spectrometry analyses identified S137 of macroH2A1 linker region to be phosphorylated, for both splice variants. This modification is excluded from the Xi, but enriched in mitosis acting in chromatin condensation (Bernstein et al., 2008). Phosphorylation of the linker region, which is important for DNA interaction, might play a role in protein interactions (Muthurajan et al., 2011). Other mass spectrometry analyses observed that macroH2A1 splice variants have distinct modified sites. While macroH2A1.1 presents more T129 phosphorylation, S170 phosphorylation is enriched in macroH2A1.2, which could affect their folding, giving the role of the linker region in stabilizing heterochromatin architecture (Chakravarthy et al., 2012; Kozlowski et al., 2018; Giallongo et al., 2021). Those studies also revealed sites of acetylation (K7) or methylation (K18, K123 or K239) in macroH2A1, but none of them has been characterized for their impact in macroH2A’s central role in maintaining nuclear integrity at DNA damage sites (Chu et al., 2006; Bernstein et al., 2008; Giallongo et al., 2021). Further investigations are required to decipher whether these PTMs are important for the function of macroH2A in DNA damage signaling and DNA repair, and to ascertain whether the specific modifications of macroH2A1 splice variants contribute to its markedly different functions in regulating tumorigenicity in humans (reviewed in Hsu et al., 2021).

## Conclusion and perspectives

The large H2A family has a major impact on the DSB repair pathway choice that is finely regulated by the histone variants and the PTMs that they are decorated with. The chromatin composition at DSBs is heterogenous and dynamic to ensure the signaling orientation and maximize the repair process at the different phases of the cell cycle. Each histone variant is giving additional properties modulated by the PTMs to recruit actors of the repair process, and each isoform of variants adds another layer of fine tuning, as discussed for H2AZ1/2 or macroH2A1.1/2. Past investigations focused on the writing and reading part of the chromatin dynamics at DSBs, while the role of specific remodelers on H2A variants-containing chromatin at DSBs is still debated. Some questions on the stoichiometry of variants-containing nucleosomes at DSBs also remain. Indeed, across many studies on histone variants and their roles in DDR, few mention the possibility of heterotypic nucleosomes. As an example, H2A.X-containing nucleosomes, signaling the damage, could also contain a macroH2A1.2, promoting or modulating the downstream signaling. Similar to the “histone code” hypothesis (Strahl and Allis, 2000; Jenuwein and Allis, 2001), the “variants code” may define the different patterns of recruitment of repair machinery. Besides H2A and its variants modulating DSB repair pathway choice, modifications of the other nucleosomal histones lead to crosstalk in regulating this choice. We mentioned that H4 methylation at lysine K20 by SUV420H1 promotes NHEJ, unless H4K16 is acetylated by Tip60. While unmethylated H4K20 recruits BARD1/BRCA1 to promote HR in S/G2 (Becker et al., 2021). Interestingly, H3K36me2 appears to promote NHEJ (Fnu et al., 2011) while the H3K36me3, a mark associated with transcribed regions, seems to prioritize HR repair (Aymard et al., 2014; Clouaire and Legube, 2015).

In the model of the “access, repair, restore” proposed by G. Almouzni (Green and Almouzni, 2002; Groth et al., 2007), modified as “prime, repair, restore” (Soria et al., 2012), the chromatin state needs to be “restored” after the repair, in a process that has been linked to cell cycle checkpoint release. The dephosphorylation of H2A.X by phosphatases like PP2AC or PP4C has been described but it remains debated if this occurs directly on chromatin or after removal of H2A.XS139ph by ATP-dependent remodelers, or both (Chowdhury et al., 2005; Keogh et al., 2006; van Attikum et al., 2007; Nakada et al., 2008). The deubiquitylation of H2AK15 by the USP51 is logically another event implicated in the restoration process (Wang et al., 2016; Ai et al., 2019) as well as the action of different histone deacetylases (Aricthota et al., 2022). It is less clear if erasers like USP48 and BAP1 also play roles in restoration beside their functions in the repair process itself, while it seems logical to argue a function of BAP1 to recover local transcription after repair. In addition, the action of the deubiquitinases on histone variants was not investigated, leaving questions unanswered. Overall, additional studies are needed to enrich our knowledge on the chromatin dynamics during completion of repair and chromatin state restoration.

Aside from the H2A variants we discussed, two others H2A variants emerged as playing roles in the DDR. H2A.J sequence is highly conserved with H2A, with two additional serines and one tyrosine while conserving lysines 125, 127 and 129 at the C-terminus (Tanaka et al., 2020). The SQ*KTKSK sequence is potentially phosphorylated as in the H2A.X variant. Moreover, similar to the C-terminal lysines in H2A being ubiquitylated by the BARD1-BRCA1 complex, it is likely that H2A.J is also modified by the complex. The H2A.J variant has been associated with the persistent DNA damages and appears to be correlated with senescence-associated secretory phenotype (Contrepois et al., 2017; Isermann et al., 2020). Another distant H2A variant, known as H2A.B (or H2A.Bbd), differs from canonical H2A with only 48% identity (González-Romero et al., 2008). In contrast to macroH2A, H2A.B is termed Barr body deficient (Bbd) because it is absent from the inactive X chromosome in females. It shows a preference for binding to regions with hyperacetylated H4, indicating enrichment in active genomic regions and implying a role in gene regulation (Chadwick and Willard, 2001; Ishibashi et al., 2010). Nucleosomes containing H2A.B organize DNA less tightly and exhibit lower stability compared to canonical nucleosomes, resulting in a more relaxed and elongated structure (Arimura et al., 2013; Sansoni et al., 2014). Studies have uncovered potential roles of H2A.B in DNA repair. Indeed, H2A.B was found, through proteomic analysis, to transiently localize to sites of DNA synthesis during replication and DNA repair, potentially affecting cell cycle regulation and DNA damage sensitivity. Cells overexpressing H2A.B have a shortened S phase and are more prone to DNA damages, as observed in Hodgkin’s lymphoma cells aberrantly expressing this variant (Sansoni et al., 2014).

Many cancers and pathologies involve histone H2A and its variants, including modifications that interact with specific readers after DNA double-strand break. Examples include ataxia telangiectasia and the RIDDLE syndrome linked with immunodeficiencies and increased risks of cancer development (McKinnon, 2004; Stewart et al., 2009; Lai and Chan, 2024). Improper or defective repair pathway in a particular cell context could be catastrophic. Thus, better understanding the regulation of the signals and the modulation of actors during the repair pathway orientation is a continuing challenge to address.
